# Supplementary material for: Aortic pathology from protein kinase G activation is prevented by an antioxidant vitamin B12 analog
Source: Nat Commun. 2019 Aug 6;10:3533. doi: 10.1038/s41467-019-11389-1 (PMC6684604; doi:10.1038/s41467-019-11389-1)
Supplement: Supplementary file 1 — Supplementary Information [file 41467_2019_11389_MOESM1_ESM.pdf]

SUPPLEMENTARY MATERIALS FOR:

Aortic Pathology from Protein Kinase G Activation Is Prevented by an Antioxidant Vitamin B_{12} Analog

(Schwaerzer, et al.)

Suppl. Fig. 1

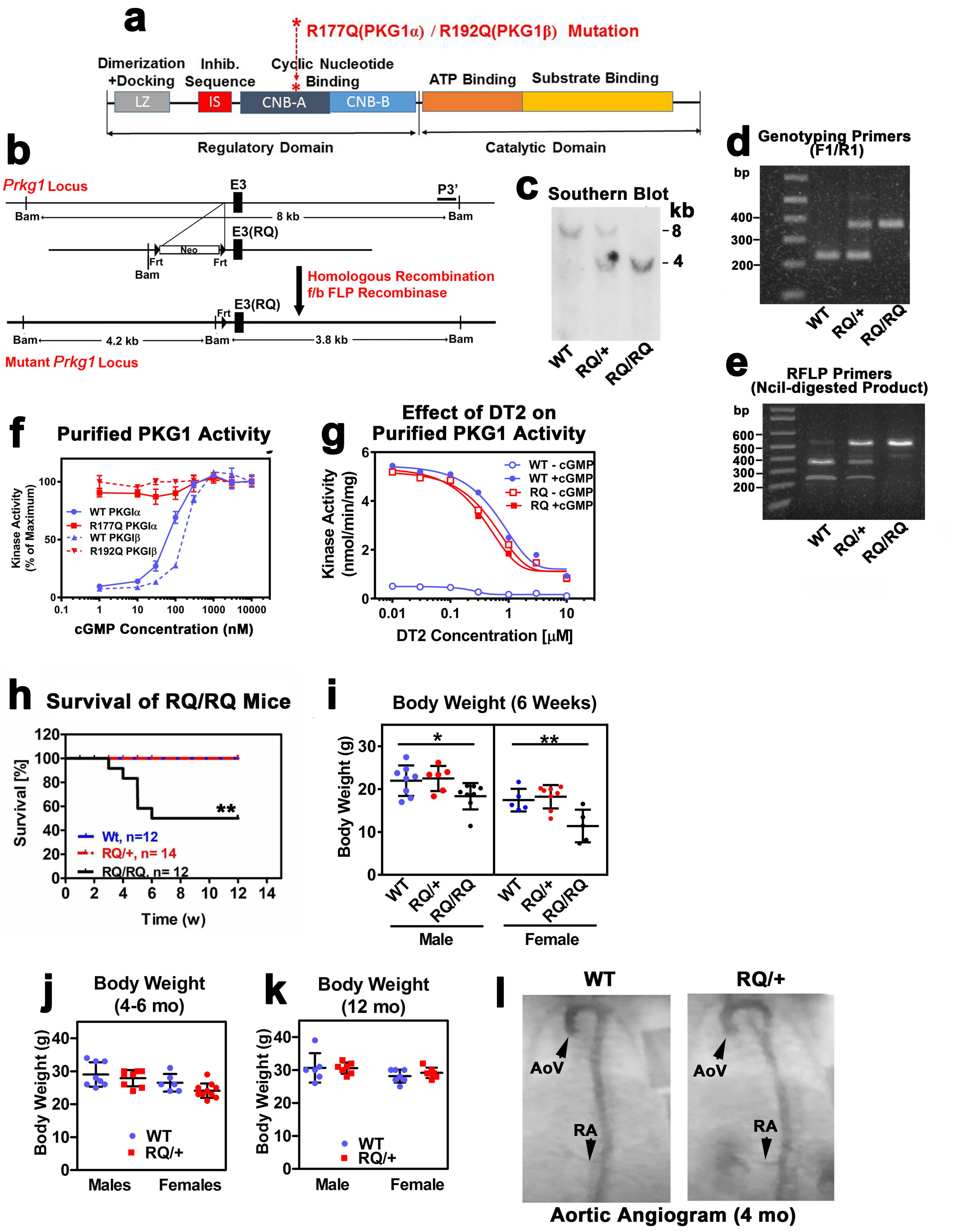
a
R177Q(PKG1α) / R192Q(PKG1β) Mutation
Dimerization +Docking
Inhib. Sequence
Cyclic Nucleotide Binding
ATP Binding
Substrate Binding
LZ
IS
CNB-A
CNB-B
Regulatory Domain
Catalytic Domain
b
Prkg1 Locus
E3
P3'
Bam
8 kb
E3(RQ)
Neo
Frt
Homologous Recombination f/b FLP Recombinase
4.2 kb
3.8 kb
Mutant Prkg1 Locus
c
Southern Blot
kb
8
4
WT
RQ/+
RQ/RQ
d
Genotyping Primers (F1/R1)
bp
400
300
200
e
RFLP Primers (Ncil-digested Product)
600
500
f
Purified PKG1 Activity
Kinase Activity (% of Maximum)
WT PKGIα
R177Q PKGIα
WT PKGIβ
R192Q PKGIβ
cGMP Concentration (nM)
g
Effect of DT2 on Purified PKG1 Activity
Kinase Activity (nmol/min/mg)
WT - cGMP
WT +cGMP
RQ - cGMP
RQ +cGMP
DT2 Concentration [µM]
h
Survival of RQ/RQ Mice
Survival [%]
Wt, n=12
RQ/+, n= 14
RQ/RQ, n= 12
Time (w)
i
Body Weight (6 Weeks)
Body Weight (g)
Male
Female
j
Body Weight (4-6 mo)
Males
Females
k
Body Weight (12 mo)
l
AoV
RA
Aortic Angiogram (4 mo)

<u>Supplementary Figure 1:</u> Generation of PKG1$^{RQ/+}$ and PKG1$^{RQ/RQ}$ mice. **(a)** PKG1α and PKG1β are derived from the same gene (*Prkg1*) by differential splicing and differ only in their first ~ 100 amino acids comprising the leucine zipper dimerization/docking domain (LZ) and the auto-inhibitory sequence (IS). The R177Q (PKG1α) or R192Q (PKG1β) mutation is located in the first of two common cyclic nucleotide binding (CNB) domains. ATP- and substrate-binding regions of the catalytic domain are shown in orange and yellow, respectively. **(b)** Targeting construct with the RQ mutation located in exon 3 (E3) of *Prkg1*; homologous recombination and removal of the *neo* cassette with FLP recombinase generates a new BamH1 site near the mutant exon. **(c)** Southern blot of BamH1-digested genomic DNA from wild type (WT), heterozygous Prkg1$^{RQ/+}$ (RQ/+), and homozygous Prkg1$^{RQ/RQ}$ (RQ/RQ) mice (using the P3'probe indicated in panel b). **(d,e)** Genotyping of tail genomic DNA from wild type, heterozygous Prkg1$^{RQ/+}$, and homozygous Prkg1$^{RQ/RQ}$ mice using the primer pair F1/R1 flanking the remaining Frt site (d). In (e), genomic DNA was amplified with primers flanking exon 3, and the PCR product was digested with NciI; only the wild type PCR product is cut into 345 bp and 186 bp fragments (>95% digestion), because the NciI site is destroyed by the RQ mutation. **(f)** *In vitro* kinase activity of wild type (blue) and mutant (red) PKG1α (R177Q) and PKG1β (R192Q) purified from transfected 293T cells was measured with a peptide substrate in the presence of increasing cGMP concentrations. Note that both mutant enzymes show nearly completely cGMP-independent activity. **(g)** Effect of increasing concentrations of the PKG-specific inhibitory peptide DT2 on the activity of purified wild type (blue) and mutant (red) PKG1α (R177Q), measured as in panel f, but with 3 μM cGMP. **(h)** Survival of a cohort of wild type (WT, n=12), heterozygous (RQ/+, n=14), and homozygous (RQ/RQ, n=12) littermates during the first three months of life. Note the precipitous drop in survival of homozygous mice after weaning, despite the provision of liquid food (**p<0.01 for the comparison of WT and RQ/RQ mice by log-rank (Mantel-Cox) test. **(i)** Body weights of the mice shown in panel h, measured at the age of 6 weeks (means ± SD, *p<0.05 and **p<0.01 for the indicated comparisons). **(j,k)** Body weights of male and female mice at the ages of 4- to 6-months and 12-months; mice are the same as shown in Fig. 1f-h and Suppl. Fig. 2i . **(l)** Aortic angiography in 4-month-old *Prkg1*$^{RQ/+}$ mice shows no gross developmental abnormalities (AoV, level of the aortic valve; RA, renal artery).

Suppl. Fig. 2

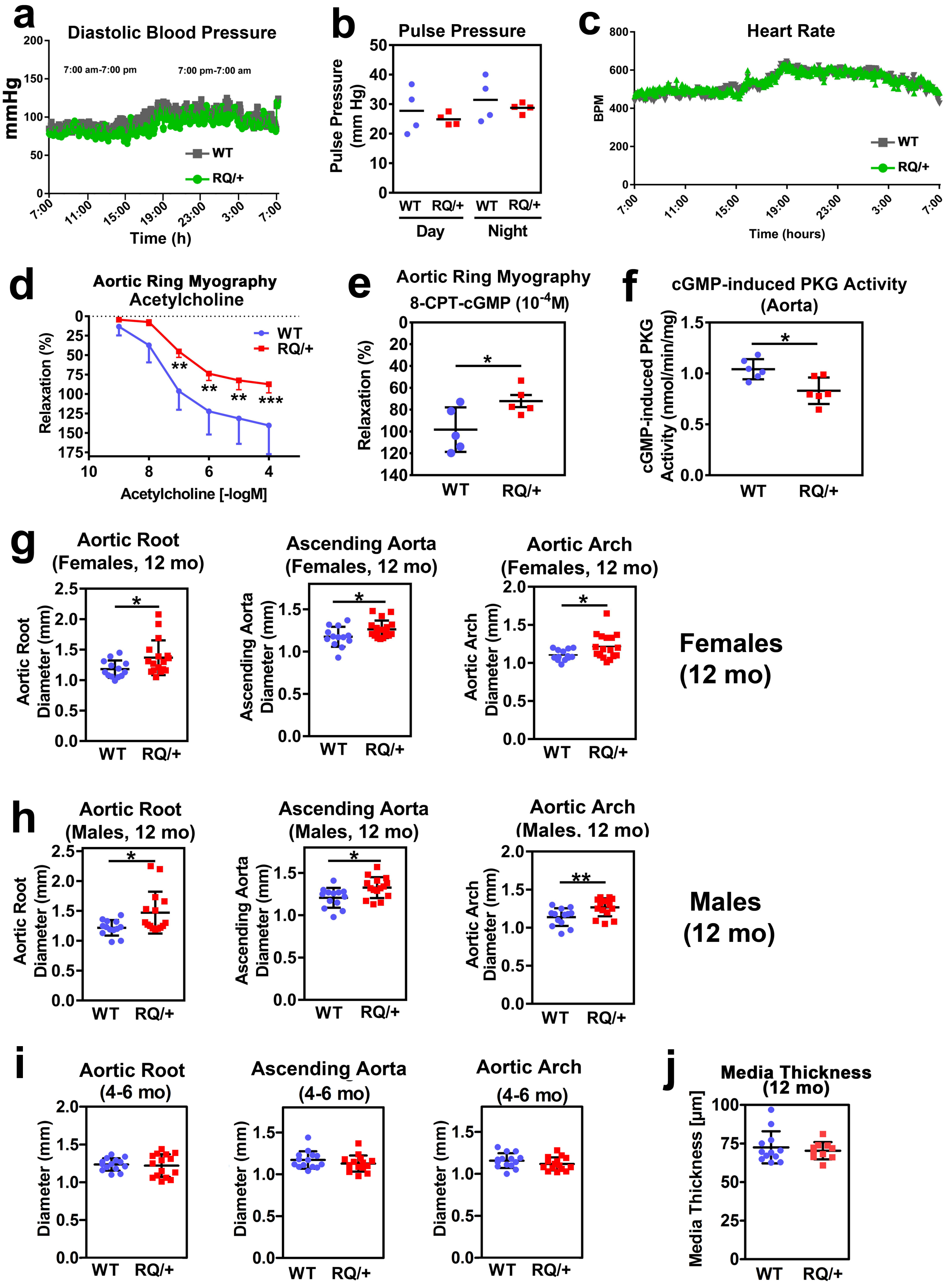

a
Diastolic Blood Pressure
7:00 am-7:00 pm
7:00 pm-7:00 am
mmHg
Time (h)
WT
RQ/+
b
Pulse Pressure
Pulse Pressure (mm Hg)
WT RQ/+ WT RQ/+
Day
Night
c
Heart Rate
BPM
Time (hours)
WT
RQ/+
d
Aortic Ring Myography
Acetylcholine
Relaxation (%)
Acetylcholine [-logM]
WT
RQ/+
e
Aortic Ring Myography
8-CPT-cGMP (10^-4M)
Relaxation (%)
WT RQ/+
f
cGMP-induced PKG Activity
(Aorta)
cGMP-induced PKG Activity (nmol/min/mg)
WT RQ/+
g
Aortic Root
(Females, 12 mo)
Aortic Root Diameter (mm)
Ascending Aorta
(Females, 12 mo)
Ascending Aorta Diameter (mm)
Aortic Arch
(Females, 12 mo)
Aortic Arch Diameter (mm)
Females
(12 mo)
h
Aortic Root
(Males, 12 mo)
Ascending Aorta
(Males, 12 mo)
Aortic Arch
(Males, 12 mo)
Males
(12 mo)
i
Aortic Root
(4-6 mo)
Ascending Aorta
(4-6 mo)
Aortic Arch
(4-6 mo)
Diameter (mm)
j
Media Thickness
(12 mo)
Media Thickness [µm]
WT RQ/+

<u>Supplementary Figure 2:</u> **Telemetric blood pressure, ultrasound measurements of aortic diameters, and aortic ring myography in heterozygous *Prkg1*$^{RQ/+}$ mice compared to wild type (WT) littermates.** **(a-c)** Diastolic blood pressure and heart rate measured by telemetry over 24 h in 4-month-old WT and *Prkg1*$^{RQ/+}$ male mice; pulse pressure was average during rest (7 am – 7 pm) and activity (7pm-7am) periods (n=4 per genotype).). (**d,e**) Isometric tension measurements performed in aortic rings of 8 month-old WT and *Prkg1*$^{RQ/+}$ female mice (n=5 per genotype) after pre-contraction to 0.15g with prostaglandin F2α. Dose-response curve for acetylcholine-induced relaxation (d), and % relaxation in response to 100 μM 8-(4-chorophenylthio)cGMP (e). (**f**) cGMP-induced PKG activity in aortic extracts was calculated as the difference between activity in the presence and absence of 3 μM cGMP (derived from Fig. 1a). **(g-i)** Diameters of the thoracic aorta measured by ultrasound as described in Fig. 1e,f. For separate analysis of 12 month-old females (g) and males (h), the 7 female and 6 male animals shown in Fig. 1f were combined with untreated animals shown in Fig. 4d (7 WT and 11 RQ/+ females; and 7 WT and 9 RQ/+ males). Panel i shows 4- to 6-month-old WT and *Prkg1*$^{RQ/+}$ mice of both genders (WT: n= 6M + 7F and *Prkg1*$^{RQ/+}$ mice: n= 7M+ 8F). **(j)** Media thickness measured in 12-month-old mice of both genders, using hematoxylin- and Van Giessen-stained cross-sections of the ascending aorta (n= 7M + 6F per genotype). *p<0.05 and **p<0.01 by two-sided t-test (panel f), by Mann-Whitney test (panels e,g,h), or by two-way ANOVA (panel d).

Suppl. Fig. 3

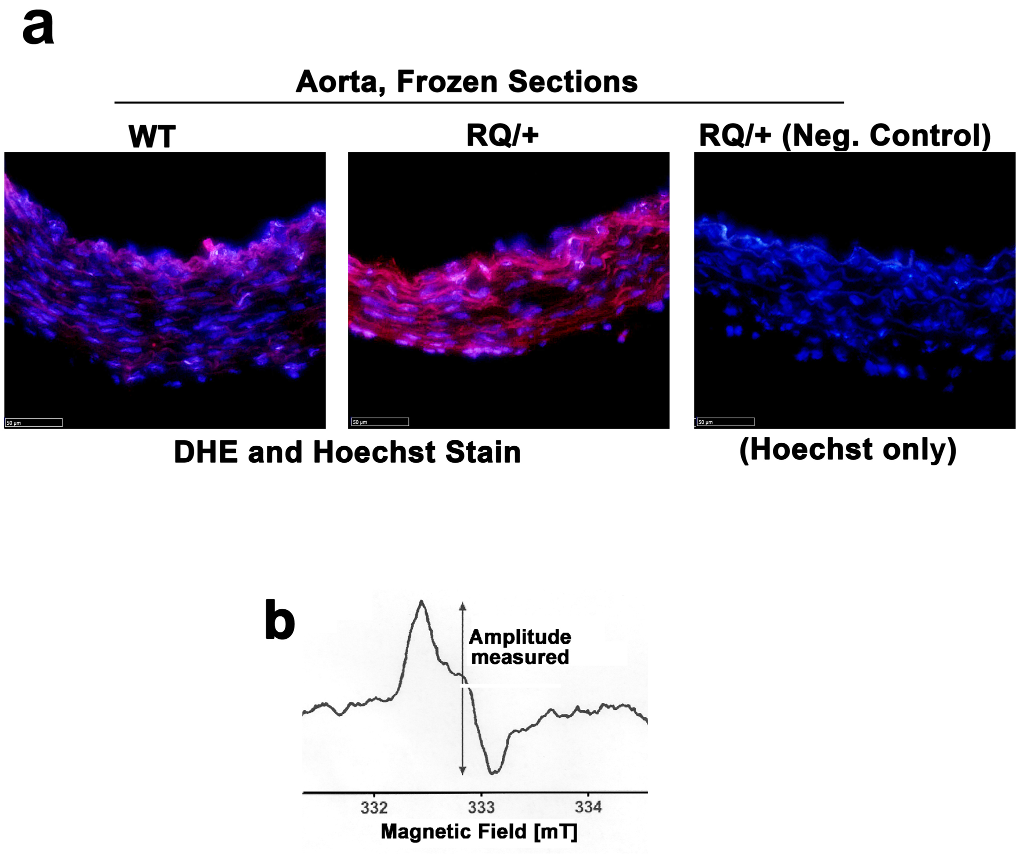

<u>Supplementary Figure 3:</u> **Increased oxidative stress in the aorta of 12-month-old heterozygous *Prkg1*$^{RQ/+}$ mice. (a)** Representative fluorescent images of frozen sections from aortas stained with dihydroethidium (DHE). Images are representative of sections from n=4 mice per genotype. **(b)** Example of the electron paramagnetic resonance signal obtained for ascorbyl radical in mouse serum, as shown in Fig. 2f.

Suppl. Fig. 4

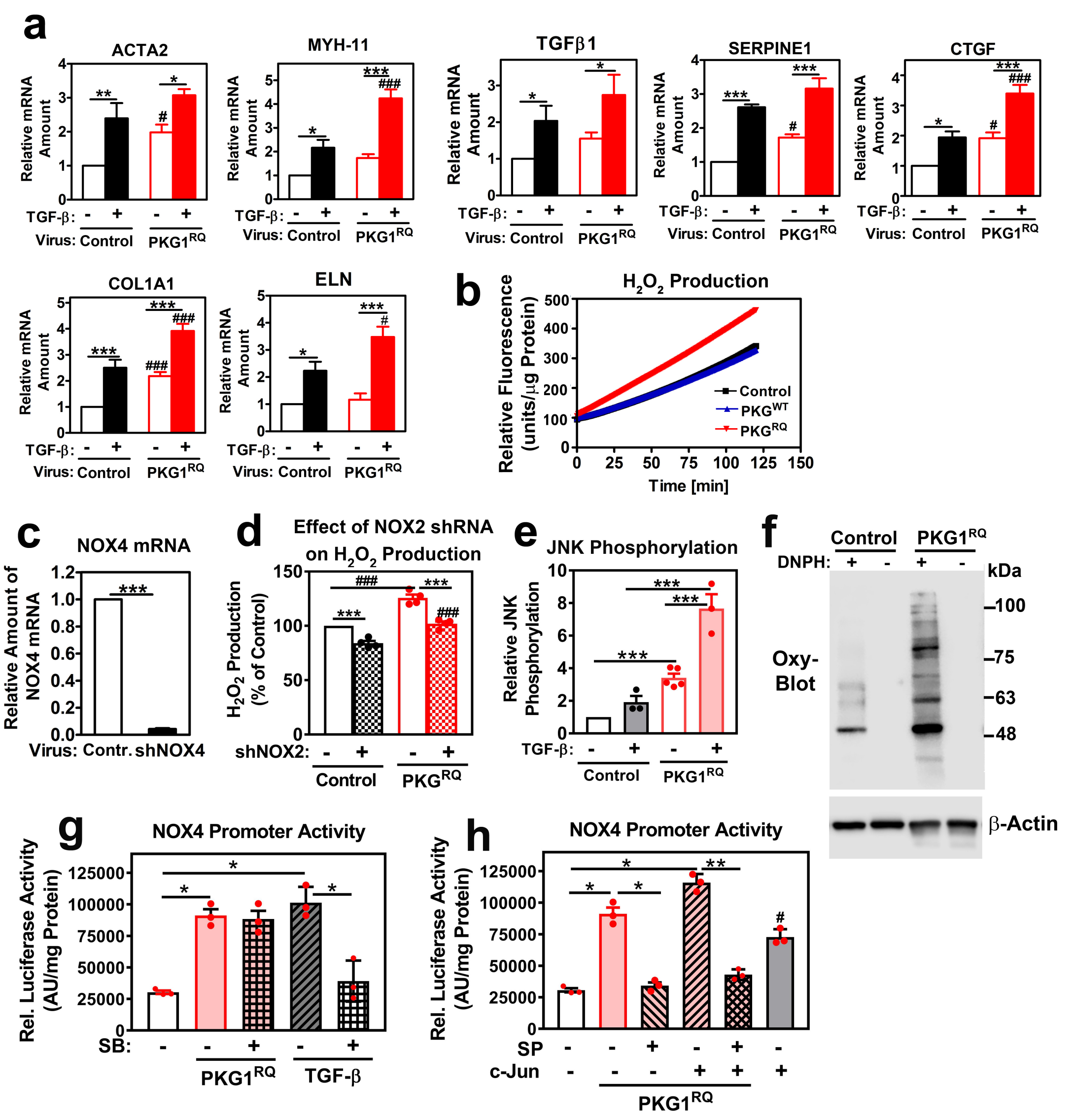

Supplementary Figure 4: Altered gene expression, increased H_2O_2 production, JNK phosphorylation, and protein oxidation in human SMCs expressing PKG1^{RQ}; effect of PKG1^{RQ} on NOX4 promoter activity. **(a)** Effect of TGF-β treatment (24 h) on relative mRNA expression of contractile markers (*ACTA2, MYH11*) and TGF-β-related genes (*TGFB1, SERPINE1, CTGF, COL1A1*, and *ELN*) in SMCs expressing PKG1^{RQ}, norma-lized to phosphoglycero-kinase-1 mRNA and compared to cells infected with control virus. **(b)** H_2O_2 production measured by Amplex Red fluorescence in human aortic SMCs infected with virus encoding GFP (control, black), wild type PKG1 (blue) or mutant PKG1^{RQ} (red); cells were re-plated 48 h after infection at a density of 2 x10^4 cells/cm^2, and Amplex Red was added 16 h later. **(c)** *NOX4* mRNA knockdown by *NOX4*-specific shRNA, measured by RT-PCR in human SMCs. **(d)** Effect of *NOX2*-specific shRNA on H_2O_2 production in human SMCs infected with control or PKG1^{RQ} virus. *NOX2* mRNA knockdown could not be reliably determined, because the amount of NOX2 mRNA in control SMCs was at the limit of detection. **(e)** JNK activation assessed in control and PKG1^{RQ}-expressing SMCs by Western blotting with phospho-specific antibody, as shown in Fig. 3h; some cells were treated with TGF-β for 2 h. **(f)** Protein aldehyde groups in control and PKG1^{RQ}–expressing human SMCs were assessed by OxyBlot™ as described in Fig. 2g. **(g,h)** Murine 10T1/2 mesenchymal cells were co-transfected with expression vector encoding PKG1^{RQ}, c-Jun, or empty vector, and a luciferase reporter under control of the murine *Nox4* promoter. Cells were treated with vehicle (0.1% DMSO), TGF-β (3 ng/ml), the TGF-β receptor-1 inhibitor SB505124 (3 μM), or the JNK inhibitor (25 μM), as indicated. Luciferase activity was measured 24 h later and normalized to protein concentration. The graphs show means ± SEM of at least three independent experiments; *$p<0.05$, **$p<0.01$, ***$p<0.001$ for the indicated comparisons, and $^{\#}p<0.05$, $^{\#\#\#}p<0.001$ for the comparison between SMCs expressing PKG1^{RQ} (or c-Jun) *versus* control cells receiving the same treatment. Panel c, two-sided t-test; panels g,h one-way ANOVA; panels a,d,e two-way ANOVA.

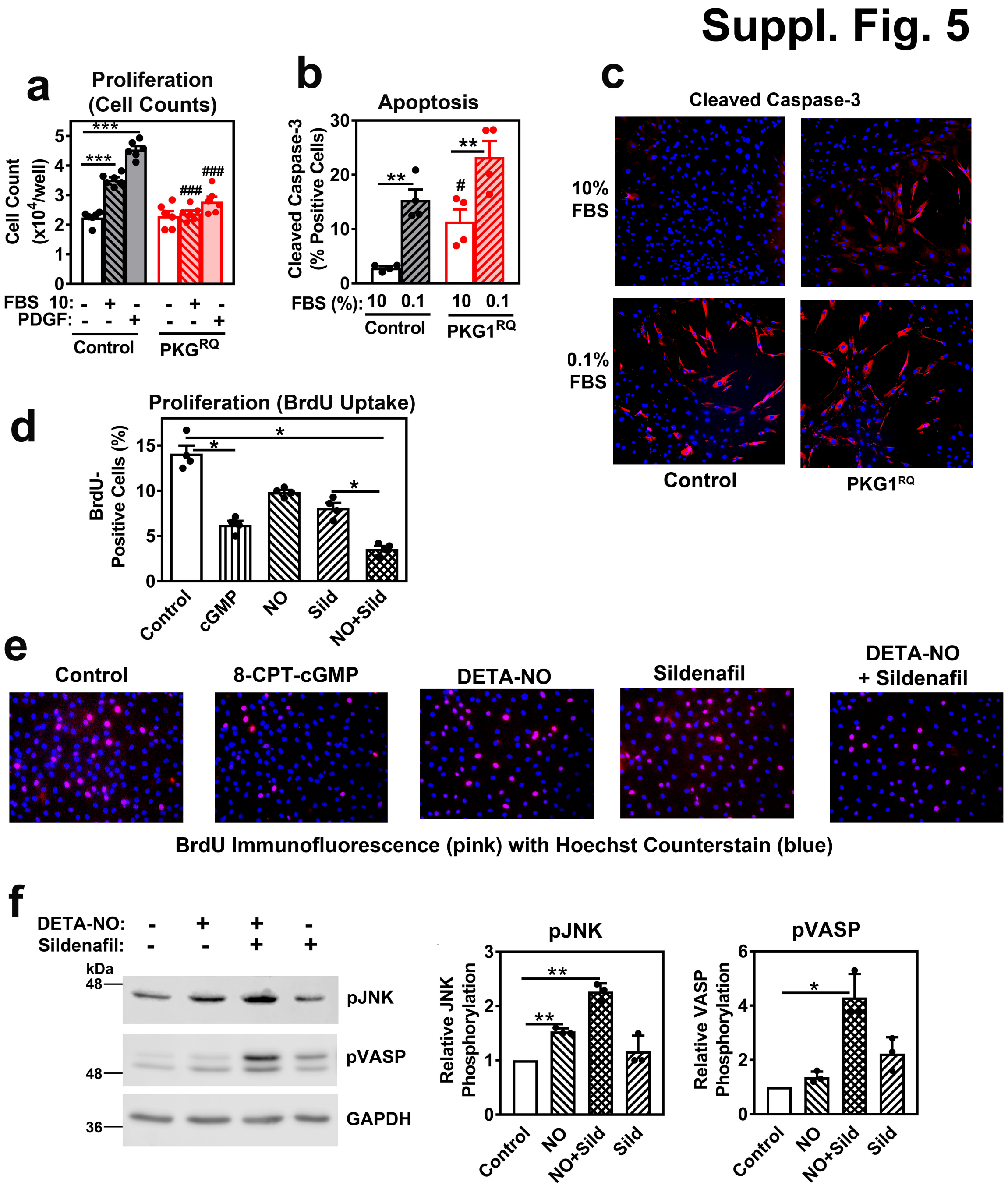

<u>Supplementary Fig. 5:</u> Decreased Proliferation and increased apoptosis in human SMCs expressing PKG1^{RQ}; effect of NO/sildenafil-mediated activation of endogenous PKG1 on proliferation and JNK activity. (a-c) Human SMCs were infected with control virus or virus expressing PKG1^{RQ}. Fourty-eight hours later, cell proliferation was measured in the absence and presence of fetal bovine serum, (FBS, 10%) or platelet-derived growth factor (PDGF, 30 ng/ml) by counting cells 72 h after re-plating (a). Apoptosis was assessed by cleaved caspase-3 immunofluorescence staining of cells cultured in the presence of 10% FBS or 0.1% FBS for 24 h (b), with representative images shown (c). **(d,e)** SMCs were cultured in media containing 10% FBS and were treated with 8-CPT-cGMP (100 μM), the NO-donor DETA-NONOate (3 μM), the PDE5 inhibitor sildenafil (100 μM) or both agents for 48 h, and BrdU uptake into S-phase cells was determined as described in Fig. 3l. **(f)** After 12 h in media with 0.5% FBS, cells were treated for 10 min with the agents described in panel d, and JNK and VASP phosphorylation were assessed by Western blotting with phospho-specific antibodies as described in Fig. 3h and 3b, respectively. Blots were quantified using LI-COR Odyssey with ImageStudio, V5. Graphs show means ± SEM of at least three independent experiments; $^{*}p<0.05$, $^{**}p<0.01$, $^{***}p<0.001$ for the indicated comparisons, and $^{\#}p<0.05$ and $^{\#\#\#}p<0.001$ for the comparison between SMCs expressing PKG1^{RQ} *versus* control cells receiving the same treatment (two-way ANOVA for panels a,b and one-way ANOVA for panels d,f).

Suppl. Fig. 6

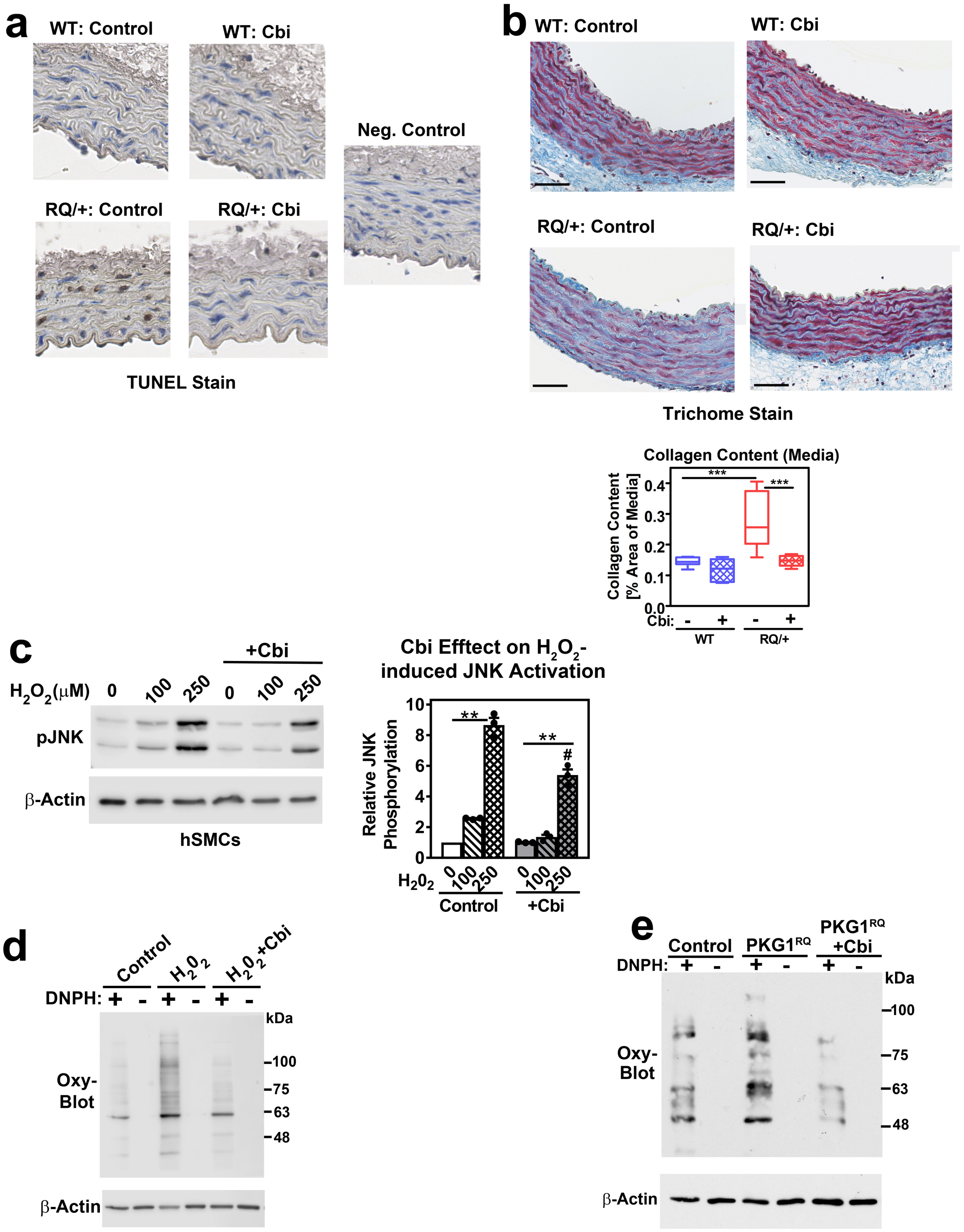

Supplementary Figure 6: Effects of Cbi on oxidative stress markers in SMCs *in vitro* and on apoptosis and collagen content in the aorta of *Prkg1*$^{RQ/+}$ mice. **(a,b)** Mice were treated with cobinamide (Cbi) as described in Fig. 4. SMC apoptosis was assessed by TUNEL staining (a, brown nuclei) as in Fig. 4c (80x). Collagen content (b, blue) of the media was quantified by ImagePro on Masson-Trichrome-stained cross-sections of the ascending aorta (40x with bar 50 μm; n=6-7 mice per group). (c) Human aortic SMCs were treated for 1h with H_2O_2 (100 or 250 μM); some cells were pre-treated with Cbi (100 μM) for 1 h. JNK activation was assessed on Western blots using a phospho-specific antibody. In the bar graph, JNK phosphorylation was quantified by ImageJ and normalized to β-actin, with untreated control cells assigned a value of one (means ±SEM of three independent experiments). **(d)** Protein oxidation was assessed in SMCs treated with 100 μM H_2O_2 for 16 h in the absence or presence of Cbi. Protein carbonyl groups were detected by OxyBlot^{TM} after derivatization with 2,4-dinitrophenylhydrazine (DNPH); non-derivatized extracts served as control, blots were reprobed with a β-actin antibody. **(e)** SMCs were infected with control virus or virus encoding PKG1^{RQ} as described in Fig. 3j, some cells were treated with 10 μM Cbi for 48h. Protein oxidation was measured as in panel d. **$p<0.01$, ***$p<0.001$ for the indicated comparisons; $^{\#}p<0.05$ for the comparison between absence *versus* presence of Cbi, by 2-way ANOVA.

Suppl. Fig. 7

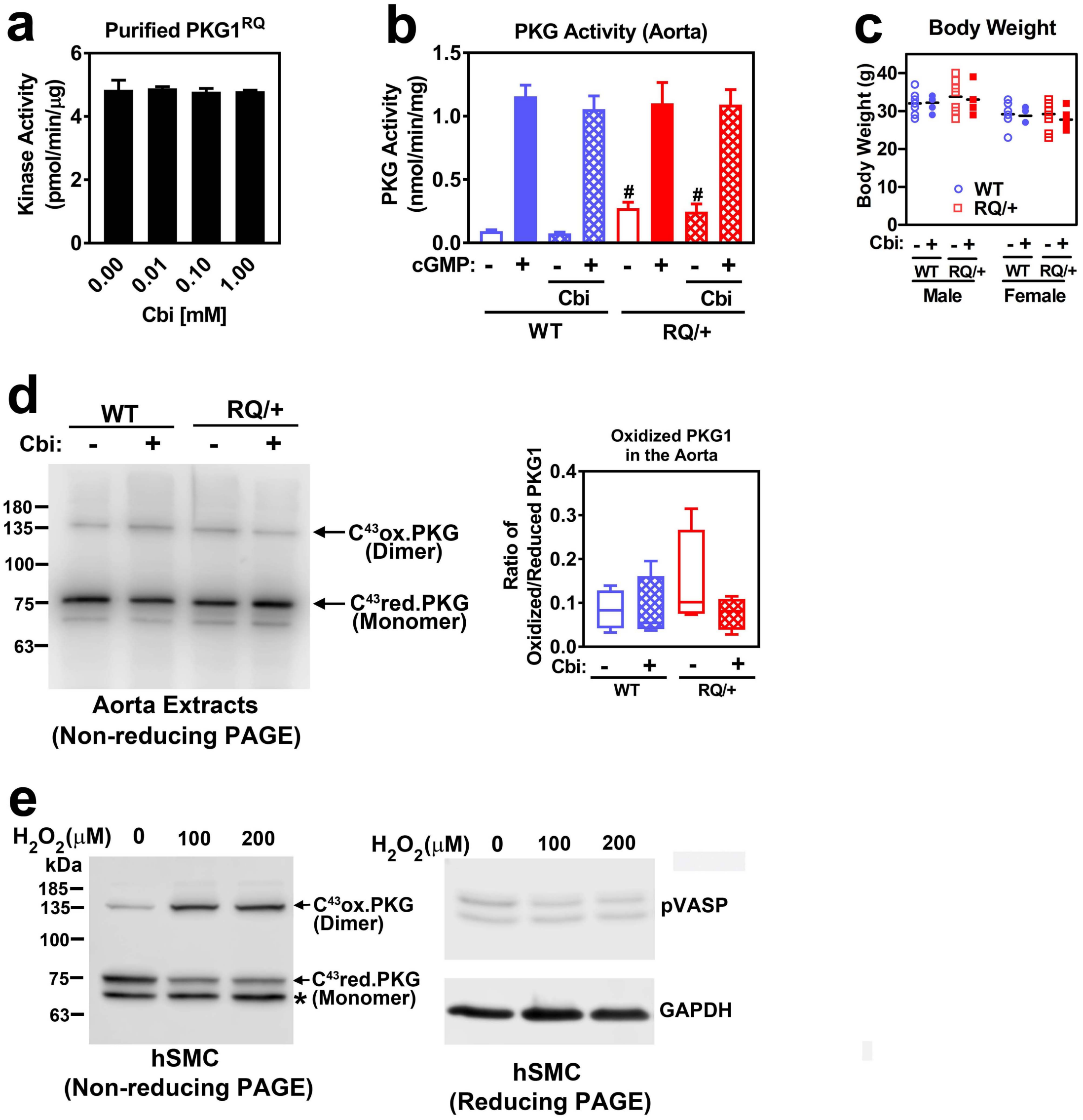

Supplementary Figure 7: Effect of Cobinamide and H_2O_2 on PKG activity and PKG oxidation. (a) Lack of effect of increasing Cbi concentrations on the basal activity of purified PKG1α (R177Q), measured as in Suppl. Fig. 1f, but in the absence of cGMP. **(b)** Eleven-month-old wild type and $PKG1^{RQ/+}$ mice were treated for one month with Cbi (1 mM) in the drinking water or were left untreated, and PKG activity was measured in aortic extracts in the absence and presence of cGMP, as described in Fig. 1a (n= 5 female mice per group; #$p<0.05$ for the comparison to basal PKG activity in untreated wild type mice, by 2-way ANOVA). **(c)** Body weight of 12-month-old mice described in Fig. 4, with males and females shown separately; some mice were treated with Cbi for 6 months, as described in Fig. 4. **(d)** Oxidation of Cys^{43} in PKG1α assessed by gel shift assay under non-reducing conditions, with the cross-linked PKG1α dimer representing Cys^{43}-oxidized enzyme.[4] The ratio of oxidized (dimeric)/reduced (monomeric) PKG1 present in aortic extracts from wild type and $PKG1^{RQ/+}$ mice was measured using LI-COR Odyssey with ImageStudio, V5. Some mice were treated with Cbi for one month as described in panel b (n= 4 female mice per group). (e) Human SMCs were treated for 1 h with 100-200 µM H_2O_2 or were left untreated as indicated. Cell extracts were analysed by SDS-PAGE either under non-reducing conditions to determine Cys^{43} oxidation in PKG1α as described in panel d, or under regular, reducing conditions to assess VASP phosphorylation as a measure of PKG activity. Note that PKG activity is not increased by H_2O_2 despite an increase in the amount of oxidized PKG dimer. GAPDH served as a loading control, and the asterix indicates a contaminating band below the reduced PKG monomer.

Suppl. Fig. 8

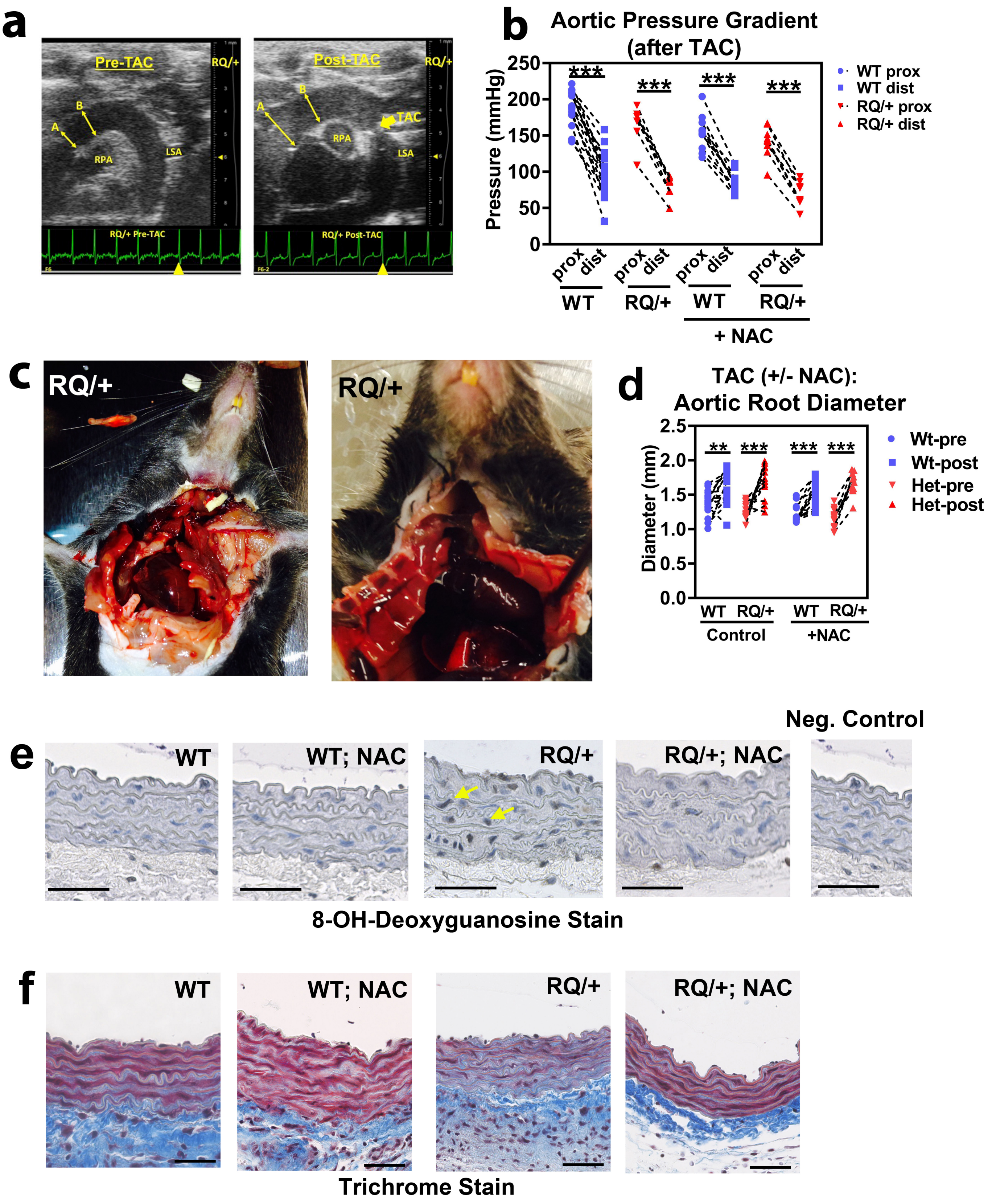

a
Pre-TAC
Post-TAC
RQ/+
A
B
RPA
LSA
TAC
RQ/+ Pre-TAC
RQ/+ Post-TAC
b
Aortic Pressure Gradient
(after TAC)
Pressure (mmHg)
250
200
150
100
50
0
***
prox
dist
WT
RQ/+
+ NAC
WT prox
WT dist
RQ/+ prox
RQ/+ dist
c
RQ/+
d
TAC (+/- NAC):
Aortic Root Diameter
Diameter (mm)
2.5
2.0
1.5
1.0
0.5
0.0
**
***
Control
+NAC
Wt-pre
Wt-post
Het-pre
Het-post
e
WT
WT; NAC
RQ/+
RQ/+; NAC
Neg. Control
8-OH-Deoxyguanosine Stain
f
WT
WT; NAC
RQ/+
RQ/+; NAC
Trichrome Stain

__Supplementary Figure 8__: **Aortic pressure gradients, aortic dilation, and hemothorax after TAC; effect of NAC on DNA oxidation and media collagen content.** **(a)** Aortic arch shown by ultrasound in *Prkg1*$^{RQ/+}$ mice before and 2 weeks after TAC surgery, using the same settings as shown for wild type mice in Fig. 5a (A=aortic root and B=ascending aorta; RPA, right pulmonary artery; LSA, left subclavian artery). **(b)** Invasive aortic blood pressure measurement 2 weeks after TAC in the ascending aorta (proximal) and descending aorta (distal to the constriction); some mice were treated with NAC as described in Fig. 5 (male mice only: n=17 WT untreated, n=8 *Prkg1*$^{RQ/+}$ untreated, n=11 WT treated with NAC, n=9 *Prkg1*$^{RQ/+}$ treated with NAC; ***p<0.001 for the indicated comparisons by 2-way ANOVA). **(c)** Representative images of PKG1$^{RQ/+}$ mice that died from aortic rupture within two weeks after TAC surgery: hemothorax on the left, hemothorax and hemopericardium on the right. **(d)** Ultrasound measurements of the aortic root diameter in WT and *Prkg1*$^{RQ/+}$ mice before and after TAC surgery (only mice surviving 14 d post TAC were included, as described in Fig. 5c; ***p<0.001 for the indicated comparisons by 2-way ANOVA). **(e)** Representative 8-OH-deoxyguanosine immunohistochemical stains of ascending aorta sections from the animals described in Fig. 5h (bars 50 µm, arrows show some examples of brown nuclei counted as positive). **(f)** Representative Masson-Trichrome stains of cross-sections of ascending aortas from the animals described in Fig. 5i, used to measure media collagen content (bars 50 µm).

SUPPL. FIG. 9

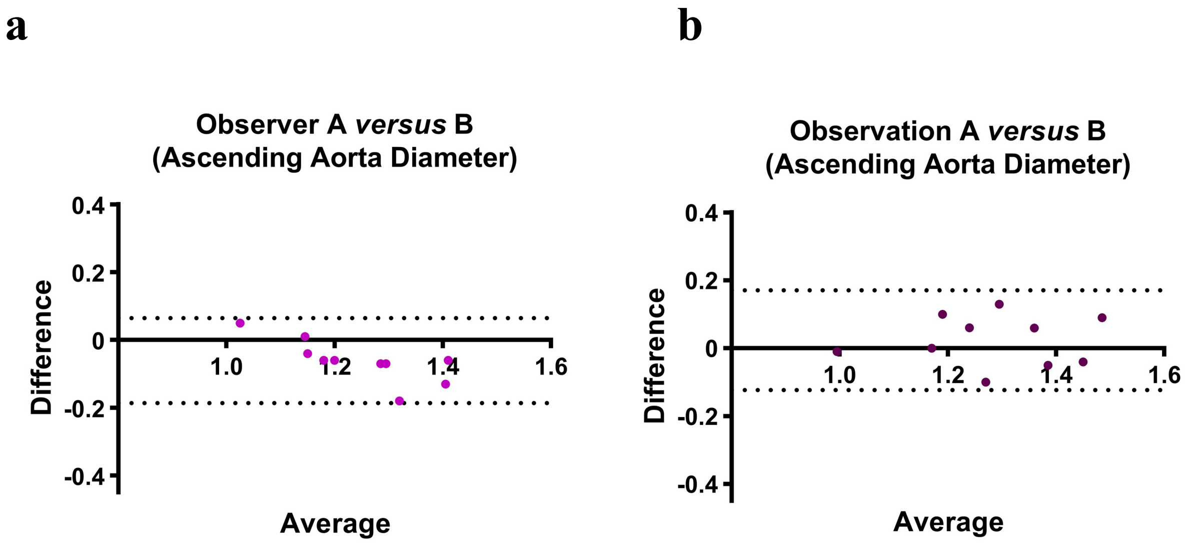

__Supplementary Figure 9__: **Inter- and intra-observer variations between aortic ultrasound measurements.** Repeated measurements of ascending aortic diameters were performed by two independent observers (a) or by a single, highly experienced operator (b). Bland Altman analyses were performed to determine inter- and intra-observer variability of echo measurements. Measuring the diameter of the ascending aorta, the average of the differences between two observers was -0.061 mm (with 95% limits of agreement between -0.18 and 0.06 mm), and the average of differences between two observations by the same observer were 0.024 mm (with 95% limits of agreement between -0.12 and 0.17 mm).

Supplementary Table 1. Blood Chemistry

Eleven month old wild type and RQ/+ mice received 1 mM cobinamide in their drinking water or were left untreated for one month (n =5 female mice for each group). Mice were euthanized with an overdose of ketamine and xylazine, and blood was obtained by cardiac puncture. The blood urea nitrogen, creatinine, albumin, alanine transaminase, alkaline phosphatase, and total bilirubin were measured in a VetScan2 analyzer. Homocysteine was measured spectrophotometrically using a kit from Crystal Chem, Inc., and methylmalonic acid was measured by ELISA using a kit from Abbexa, Ltd. For all blood components, there was no significant difference by a two-tailed t test between wild type and RQ/+ samples, and between untreated and cobinamide-treated samples.

	**Untreated**		**Cobinamide-treated**	
**Component**	**Wild type**	**RQ/+**	**Wild type**	**RQ/+**
Blood urea nitrogen	21 ± 1.1	24 ± 7.8	19 ± 2.5	20 ± 1.9
Creatinine	0.3 ± 0.05	0.4 ± 0.1	0.2 ± 0.06	0.3 ± 0.06
Albumin	3.6 ± 0.6	4.2 ± 0.2	3.8 ± 0.2	3.7 ± 0.2
Alanine transaminase	34 ± 15	33 ± 5	120 ± 89	52 ± 28
Alkaline phosphatase	73 ± 35	77 ± 28	57 ± 38	47 ± 9
Total bilirubin	0.3 ± 0.02	0.3 ± 0.03	0.3 ± 0.06	0.3 ± 0.05
Homocysteine	10.1 ± 4.1	5.9 ± 4.0	9.9 ± 3.6	5.7 ± 4.8
Methylmalonic acid	38.9 ± 15.8	28.8 ± 7.4	33.4 ± 8.3	27.3 ± 9.7

Supplementary Table 2. Complete Blood Counts

Blood obtained from the same mice described in Table 1 was analyzed on a Hemavet 950FS Multi Species Hematology System (Drew Scientific, CT). For all components, there was no significant difference by a two-tailed t test between wild type and RQ/+ samples, and between untreated and cobinamide-treated samples. n = 5 female mice for each group.

	**Untreated**		**Cobinamide-treated**	
**Component**	**Wild type**	**RQ/+**	**Wild type**	**RQ/+**
Hemoglobin	14 ± 0.5	15 ± 0.8	14 ± 1.3	14 ± 0.5
Mean corpuscular volume	53 ± 3.3	54 ± 1.2	55 ± 1.7	56 ± 0.6
White blood cell count	2.2 ± 1.3	1.7 ± 0.5	2.3 ± 0.6	3.0 ± 0.8
Platelet count	427 ± 67	478 ± 71	304 ± 197	465 ± 57

Supplementary Table 3: Reagents

Name of the chemical	Source	Catalog number	Working Concentration
SP600125	Cayman Chemical	10010466	10 mM
SB505124	Cayman Chemical	11793	3 µM
DETA NONOate	Cayman Chemical	82120	3 µM
GKT 137831	Selleck Chem	S7171	15 µM
Sildenafil	Millipore-Sigma	10008671	100 µM
5-Bromo-2'-deoxyuridine	Millipore-Sigma	B5002	200 µM
Transforming growth factor-β1	Millipore-Sigma	GF439	3 ng/ml
DT-2	Sigma	D0444	2-10 µM
8-(4-chlorophenylthio)-cGMP (8-pCPT-cGMP)	Biolog	C009	100 µM
Prostaglandin F2a	Thermo-Fischer	AAJ67245LB0	2-8 µM

Supplementary Table 4: Antibodies

Antibodies	Company	Cat. #	Lot #
PKG-1 (C8A4) rabbit monoclonal	Cell Signaling Technology	#3248	4
Phospho-VASP (Ser239)	Cell Signaling Technology	#3114	6
Phospho-SAPK/JNK (Thr183/Tyr185)	Cell Signaling Technology	#9251	24
β-Actin (C-4) antibody-HRP-conjugate	Santa Cruz Biotechnology	sc-47778	F0215
Anti-8-hydroxy-deoxyguanosine Ab (Clone # 7D7E4)	Abcam	ab26842	

Supplementary Table 5: Murine Primers Used for qRT-PCR

Name of Primer (murine)	Sequence – Forward (5'-3')	Sequence – Reverse (5'-3')
18S-rRNA	GAT CCA TTG GAG GGC AAG TCT	CCA AGA TCC AAC TAC GAG CTT TTT
Collagen1-alpha1 (*Col1a1*)	CTG CTG GCA AAG ATG GAG A	ACC AGG AAG ACC CTG GAA TC
Collagen3-alpha1 (*Col3a1*)	AGG CTG AAG GAA ACA GCA AA	AAA AAG CAA ACA GGG CCA AT
Connective tissue growth factor (*Ctgf*)	CCT GGT CCA GAC CAC AGA GT	GCA GCC AGA AAG CTC AAA CT
Decorin (*Dcn*)	GTC TGG CCA ATG TTC CTC AT	CTC ACA GCC GAG TAG GAA GC
Elastin (*Eln*)	TGG ACC TGG AGG ACT TGG	CAT ACT GGG CAG CCT TAG CA
Hypoxia-inducible factor-1α (*Hif1a*)	GGG GAG GAC GAT GAA CAT CAA	GGG TGG TTT CTT GTA CCC ACA
Hypoxanthine phosphoribosyl-transferase (*Hprt*)	CCA GAC AAG TTT GTT GTT GTT GGA T	GCT TTG TAT TTG GCT TTT CCA
Lumican (*Lum*)	GGC TGA TAG TGG GGT ACC TG	AAG CGC AGA TGC TTG ATC TT
Myocardin (*Myocd*)	ACC CAT GGA CTC TGC CTA TG	AGG GGT ATT GCT CAG TGG TG
Myosin Heavy chain-11 (*Myh11*)	AAG TCG CTG AAG CAA AAG GA	GCA CTC CTC CTC TGC CTC TT
NADPH oxidase-4 (*Nox4*)	ACT TTT CAT TGG GCG TCC TC	CCC CAG TAG TCT CCT AAG GTT TC
NADPH oxidase-2 (*Nox2*)	AAGACTCTGTATGGACGGCC	GCCGGATTCTGAGTTGGAGA
Plasminogen activator inhibitor-1 (*Serpine1*)	GCT CTC TGT AGC ACA GGC ACT	TGC CGA ACC ACA AAG AGA AA
Smooth muscle alpha-actin (*Acta2*)	AAT GGC TCT GGG CTC TGT AA	CTC TTG CTC TGG GCT TCA TC
Transgelin (*Tagln*)	ATG AGC CGA GAA GTG CAG TC	TCG ATC CCT CAG GAT ACA GG
Transforming growth factor-beta1 (*Tgfb1*)	GAC TTC CTG CAG CTG TTC G	GCT GAG CAT GGA AAT GGT TT
Vascular endothelial growth factor A (*Vegfa*)	GGA CAT AGA GAG AAT GAG CTT CC	CTC CGC TCT GAA CAA GGC T

Supplementary Table 6: **Sequence of Human Primers**

Name of Primer (human)	Sequence – F (5’-3’)	Sequence – R (5’-3’)
Collagen1-alpha1 (*COL1A1*)	GTGCTGTTGGTGCTAAGGGTGA	AGCACCATTGGCACCTTTAG
Connective Tissue Growth Factor (*CTGF*)	GTTCCAAGACCTGTGGGATG	GCAGCCAGAAAGCTCAAACT
Elastin (*ELN*)	CTAAAGCAGCCGCCAAAG	AACTCCACCAGGGCCAAT
Myosin Heavy chain-11 (*MYH11*)	GCCCAGGATGATGAGATGTT	ACGCCTGGTCTGTGTTTCTT
NADPH oxidase-4 (*NOX4*)	TCATGCACTGAATTCAAGACTGT	TGTCCCATATGAGTTGTTCTGG
NADPH oxidase-2 (*NOX2*)	TGAACGAATTGTACGTGGGCAG	ATCGCCAAAACCGCACCAAC
Phosphoglycerate kinase-1 (*PGK1*)	CTG TGG GGG GTA TTT GAA TGG	CTT CCA GGA GCT CCA AAC TG
Plasminogen activator inhibitor-1 (*SERPINE1*)	ACCAAGAGCCTCTCCACGTC	CAAAGAGGAAGGGTCTGTCC
Smooth muscle alpha-actin (*ACTA2*)	GTGACGAAGCACAGAGCAAA	GAGTCATTTTCTCCCGGTTG
Transforming Growth Factor beta - 1 (*TGFB1*)	CCCTACATTTGGAGCCTGGA	CGATCATGTTGGACAGCTGC
