# Supplementary material for: Aortic pathology from protein kinase G activation is prevented by an antioxidant vitamin B12 analog
Source: Nat Commun. 2019 Aug 6;10:3533. doi: 10.1038/s41467-019-11389-1 (PMC6684604; doi:10.1038/s41467-019-11389-1)
Supplement: Supplementary file 3 — Reporting Summary [file 41467_2019_11389_MOESM3_ESM.pdf]

nature research

Corresponding author(s): Renate B Pilz
Last updated by author(s): Jun 28, 2019

Reporting Summary

Nature Research wishes to improve the reproducibility of the work that we publish. This form provides structure for consistency and transparency in reporting. For further information on Nature Research policies, see Authors & Referees and the Editorial Policy Checklist.

Statistics

For all statistical analyses, confirm that the following items are present in the figure legend, table legend, main text, or Methods section.

n/a	Confirmed	
☐	☒	The exact sample size (*n*) for each experimental group/condition, given as a discrete number and unit of measurement
☐	☒	A statement on whether measurements were taken from distinct samples or whether the same sample was measured repeatedly
☐	☒	The statistical test(s) used AND whether they are one- or two-sided<br>*Only common tests should be described solely by name; describe more complex techniques in the Methods section.*
☒	☐	A description of all covariates tested
☐	☒	A description of any assumptions or corrections, such as tests of normality and adjustment for multiple comparisons
☐	☒	A full description of the statistical parameters including central tendency (e.g. means) or other basic estimates (e.g. regression coefficient) AND variation (e.g. standard deviation) or associated estimates of uncertainty (e.g. confidence intervals)
☐	☒	For null hypothesis testing, the test statistic (e.g. F, t, r) with confidence intervals, effect sizes, degrees of freedom and P value noted<br>*Give P values as exact values whenever suitable.*
☒	☐	For Bayesian analysis, information on the choice of priors and Markov chain Monte Carlo settings
☒	☐	For hierarchical and complex designs, identification of the appropriate level for tests and full reporting of outcomes
☒	☐	Estimates of effect sizes (e.g. Cohen's d, Pearson's r), indicating how they were calculated

Our web collection on statistics for biologists contains articles on many of the points above.

Software and code

Policy information about availability of computer code

Data collection	ImagePro Premiere Software (V9.0), Image J (V1.51), Li-Cor ImageStudio (V5), Digital Pathology NDP (V2)
Data analysis	GraphPad Prism 7, LabChart (AD Instruments), Li-Cor ImageStudio (V5)

For manuscripts utilizing custom algorithms or software that are central to the research but not yet described in published literature, software must be made available to editors/reviewers. We strongly encourage code deposition in a community repository (e.g. GitHub). See the Nature Research guidelines for submitting code & software for further information.

Data

Policy information about availability of data

All manuscripts must include a data availability statement. This statement should provide the following information, where applicable:

- Accession codes, unique identifiers, or web links for publicly available datasets
- A list of figures that have associated raw data
- A description of any restrictions on data availability

All relevant data supporting the findings of this study are available within the paper and its supplementary information files. The source data underlying all graphs and blots in Figs. 1-5 and Supplementary Figs, 1-8 are provided as a Source Data File. All data are available from the corresponding author upon reasonable request.

Field-specific reporting

Please select the one below that is the best fit for your research. If you are not sure, read the appropriate sections before making your selection.

☒ Life sciences ☐ Behavioural & social sciences ☐ Ecological, evolutionary & environmental sciences

October 2018

Life sciences study design

All studies must disclose on these points even when the disclosure is negative.

Sample size	A power calculation was performed based on our published ECHO data measuring differences in aortic root diameter between wild type and Acta2-deficient mice (Chen et al, Circ Res 120:1903-1915, 2017). We calculated a sample size of n=13 to detect the observed difference with a power of 0.8 (alpha=0.05).
Data exclusions	Mice dying within 24 h after TAC surgery were excluded from analyses: 6/40 wild type and 5/44 heterozygous mice (total peri-operative mortality 13%) - (described in the Methods Section)
Replication	All animal experiments were performed with male and female litter mates from multiple litters, grouped according to age. Groups of mice were analyzed at the appropriate age, and each experiment is the composite of at least two to three groups of mice analyzed independently over the period of several years. Results of in vitro experiments are the means of at least thee (mostly 4-6) independent experiments.
Randomization	At the time of weaning, male and female litter mates were separated and randomly assigned to new cages. Matched cages containing mice born within a three week period of time were randomly assigned to treatment versus vehicle groups (described in the Methods Section).
Blinding	Surgical procedures and ECHO readings were performed by highly experienced operators, who were blinded to genotype and treatment of the mice. Histomorphometric measurements were confirmed by an investigator who was blinded to the genotype and treatment group of the mice. Blinding is described in the Methods Section.

Reporting for specific materials, systems and methods

We require information from authors about some types of materials, experimental systems and methods used in many studies. Here, indicate whether each material, system or method listed is relevant to your study. If you are not sure if a list item applies to your research, read the appropriate section before selecting a response.

Materials & experimental systems

n/a	Involved in the study	
☐	☒	Antibodies
☐	☒	Eukaryotic cell lines
☒	☐	Palaeontology
☐	☒	Animals and other organisms
☒	☐	Human research participants
☒	☐	Clinical data

Methods

n/a	Involved in the study	
☒	☐	ChIP-seq
☒	☐	Flow cytometry
☒	☐	MRI-based neuroimaging

Antibodies

Antibodies used	PKG-1 (C8A4) rabbit monoclonal, Cell Signaling Technology #3248; Phospho-VASP (Ser239),Cell Signaling Technology #3114; Phospho-SAPK/JNK (Thr183/Tyr185), Cell Signaling Technology #9251 2; beta-Actin (C-4) antibody-HRP-conjugate, Santa Cruz Biotechnology #sc47778; Anti-8-hydroxy-deoxyguanosine Ab (Clone # 7D7E4), Abcam #ab26842; OxyBlotTM Protein Oxidation Detection Kit (Millipore EMD; S7150).
Validation	The PKG1-specific antibody was validated by Western blotting using cultured cells in which endogenous PKG1 was knocked down by siRNA and PKG1 was re-expressed by viral transduction, as previously published. The phospho-VASP antibody was validated in cells in which PKG1 was activated by 8-CPT-cGMP. The phospho-JNK antibody was validated in cells treated with hydrogen peroxide. All three antibodies produced bands of the appropriate molecular weight and the latter two produced the expected increase in phosphorylation signal with the appropriate stimulus. The oxyblot antibody only produced a signal after derivatization of extracts, and the signal was increased in cells treated with hydrogen peroxide, confirming validation by the manufacturer.

Eukaryotic cell lines

Policy information about cell lines

Cell line source(s)	293T cells (used for the production of recombinant PKG1) --- from the ATCC (cat # CRL3216);<br>Human primary aortic smooth muscle cells --- from Lifeline Cell Technology (Frederick, MD, cat # FC-0015)
Authentication	293T cells were obtained from the ATCC and used within 5-10 passages; human SMCs were characterized by Lifeline Cell Technology and used at passages 3-6
Mycoplasma contamination	Not tested (293T cells were previously tested by the ATCC, human aortic SMCs were tested by Lifeline Cell Technology)

Commonly misidentified lines (See ICLAC register)	293T cells were only used for the production of recombinant PKG1

Animals and other organisms

Policy information about studies involving animals; ARRIVE guidelines recommended for reporting animal research

Laboratory animals	mus musculus (laboratory mouse); almost all mice used in the experiments described were in an inbred S129/SvImJ background; only for TAC experiments, mice were back-crossed into the C57BL/6NHsd background as described in the Methods Section. The age of the mice is clearly stated in the Figure legends, and results are shown for mixed gender, unless specifically stated otherwise. The number of male and female mice in each experiment is noted in the Figure legends.
Wild animals	No wild animals included in the study
Field-collected samples	The study did not include field-collected samples
Ethics oversight	All animal experiments complied with ethical guidelines for the use of animals in research according to policies of the University of California, and were approved by the Institutional Care and Use Committee of the University of California, San Diego.

Note that full information on the approval of the study protocol must also be provided in the manuscript.
